# Self-Identity Matters: An Extended Theory of Planned Behavior to Decode Tourists’ Waste Sorting Intentions

**DOI:** 10.3390/ijerph20065099

**Published:** 2023-03-14

**Authors:** Jian Cao, Hongliang Qiu, Alastair M. Morrison

**Affiliations:** 1 School of Tourism and Foreign Languages, Tourism College of Zhejiang, Hangzhou 311231, China; 2 Postdoctoral Station of Business Administration, Fudan University, Shanghai 200433, China; 3 School of Business Administration, Tourism College of Zhejiang, Hangzhou 311231, China; 4 Zhejiang Academy of Culture & Tourism Development, Hangzhou 311231, China; 5 School of Management and Marketing, Greenwich Business School, University of Greenwich, Old Royal Naval College, Park Row, London SE10 9LS, UK

**Keywords:** theory of planned behavior, identity theory, self-identity, moral norms, tourists’ waste-sorting intentions, heritage tourism

## Abstract

Waste sorting is a practical way of handling the garbage and an effective strategy for facilitating sustainable waste management. This research extended the theory of planned behavior (TPB) with self-identity and moral norms to predict waste sorting intentions in a heritage context of tourism. A total of 403 valid self-administrated questionnaires were achieved at a heritage destination in China. The results indicated that: (1) TPB variables (i.e., attitudes toward the behavior, subjective norms, and perceived behavioral control), self-identity, and moral norms were all directly and positively linked to tourists’ waste sorting intentions, respectively; (2) self-identity indirectly influenced tourists’ waste sorting intentions through the mediation of moral norms; and (3) the integrated model exhibited better predictive utility than any single model. This research contributes to the literature on waste management in the context of tourism by extending TPB with identity and personal normative constructs. It also provides practical implications for destination managers to leverage tourists’ self-identity and moral norms for sustainable management.

## 1. Introduction

Although tourism is presumed to be a benign sector, it still has a massive impact on the environment [1,2]. To strike a balance between limited ecological systems and socio-economic benefits, considerable scholarly attention has been given to the intrinsic relationship between sustainability and tourism [3]. Among the 17 Sustainable Development Goals (SDGs) proposed by the United Nations, one goal (SDG 11) particularly addresses the need to preserve the world’s heritage resources and lessen the detrimental effects of improper waste management on the environment [4]. The swelling number of people visiting the heritage destinations following their inscription as World Heritage Site raises concerns about the environmental issue in the heritage destinations [5]. Waste mismanagement can impair the unique cultural, natural, and historical significance of the heritage sites, which disrupts the achievement of sustainable development goals [6]. Waste sorting is considered as an effective approach to mitigate the adverse influence on the environment [7]. The existing literature on this topic focuses on waste sorting behaviors in the household setting [8,9]. It is suggested that tourists traveling in an unhabitual environment may behave differently from home due to their hedonic pursuit and the short duration of their stay [10]. In this sense, the formation mechanism of waste sorting in the context of tourism may differ from the household setting. However, limited research is devoted to the waste-sorting behaviors in a context of tourism [7]. This highlights the importance and urgency of identifying the antecedents of tourists’ waste-sorting behaviors for conserving heritage sites. 

There have been mounting efforts to understand pro-environmental behaviors [10,11,12], among which substantial attempts have been made to explain the antecedents through various social psychological theories, such as the stimulus-organism-response model, the norm activation model, the cognition-affect-behavior model, reciprocity theory, theory of reasoned action and its successor, and theory of planned behavior [13,14,15,16,17]. Theory of planned behavior shows its prevalence among social cognitive theories [18]. The fundamental principle of TPB is that individuals make decisions based on the rational calculation of the costs and benefits, in which their attitudes, perceived social pressure, and control over the behavior are the key players [19]. Due to its parsimoniousness and robustness in predicting individual behaviors, TPB has been extensively applied in the research on sustainable environmental behaviors and intentions [18,20], including general pro-environmental behaviors and specific behaviors (e.g., energy saving and waste recycling) [21,22,23,24]. In terms of waste sorting, extant studies focus on the household context, rather than the context of tourism [25]. Despite the efficacy of TPB in predicting behaviors, it has also been heavily debated due to the complex nature of human behaviors [26]. It has been suggested that inclusion of other variables or theories may enhance the variance of TPB in explaining individual behaviors [27,28]. Hence, this research attempted to identify the determinants of tourists’ waste-sorting intentions by extending the TPB model with other variables.

TPB considers people performing given behaviors to be largely a psychological entity instead of a social one [29]. Accordingly, subjective norms in TPB cannot fully explain the entire spectrum of socially determined effects. Self-identity is thus considered by social researchers as another critical driver of human behaviors [26]. This identity-behavior link is developed on the premise of identity theory, which proposes that the self is comprised of a series of roles corresponding to people’s positions in their social structures [30]. When people’s particular roles are related to certain behaviors, they are more likely to perform these behaviors consistently with their self-images [30]. For example, if people consider themselves as the type of person who is concerned about the environment, there will be higher possibilities for them to take pro-environmental actions to maintain the consistency [31]. In this regard, in the context of tourism, when tourists consider themselves as eco-friendly individuals, they tend to behave more sustainably to verify the self-identity reflected in the pro-environmental behaviors. Mounting evidence demonstrates that people’s sense of identity can predict their pro-environmental behaviors or intentions [32,33]. However, research on how self-identity contributes to tourists’ waste-sorting intentions remains under researched. Consequently, self-identity is considered as a supplementary determinant of tourists’ waste sorting intentions in this research. 

To better understand the formation of tourists’ waste sorting intentions, it requires more efforts to elucidate the process through which self-identity translates into these behavioral intentions. It is noted that the adoption of most pro-environmental behaviors can be attributed to obligation-based motives, as they involve more efforts and cost but less enjoyment [34]. Specifically, the choice of pro-environmental behaviors represents a decision driven by one’s moral norms for the public good [35]. It is further suggested that an individual’s self-identity, which measures how much one views themselves as a certain type of person, affects their moral norms [36,37]. Along this line, moral norms potentially play a mediating role in this identity–behavior relationship. The effect of moral norms on one’s pro-environmental behaviors has been empirically validated in considerable research [22,38]. In addition, it has been revealed that one’s self-identity is indirectly related to people’s intentions to adopt general and specific pro-environmental behaviors through moral norms [26,39,40]. However, there is lack of empirical evidence of how tourists’ self-identity influences their waste-sorting intentions through moral norms. Accordingly, this research argued that moral norms, as another group of additional predictors of behavioral intentions, might mediate the link between tourists’ self-identity and their waste sorting intentions.

By filling the aforementioned knowledge voids, this research contributed to the research of waste sorting in the following ways. First, this research enriched the current literature by augmenting TPB with self-identity and moral norms for predicting tourists’ waste sorting intentions. Second, we identified and highlighted the direct and indirect effects of self-identity on tourists’ waste sorting intentions. Third, the current study validated the mediating role of moral norms between self-identity and tourists’ waste sorting intentions. Finally, the explanatory utility of the extended TPB framework was verified in this research. Theoretically, the additions of identity and personal normative factors into TPB shed new light on the explanation of tourists’ waste sorting intentions. Practically, findings of this research can offer some managerial implications for sustainable destination management by establishing tourists’ self-identity and enhancing their moral norms.

The rest part of this article is organized as follows. Section 2 provides an overview of the theoretical framework and formulates relevant hypotheses based on the review of the existing literature. Then, the research design and the data analysis are presented in Section 3 and 4, respectively. Section 5 offers a comprehensive discussion of the main findings and their theoretical and practical implications. The last part, Section 6, acknowledges the potential limitations of this study and proposes some directions for future research.

## 2. Literature Review and Hypotheses Development

### 2.1. Waste Sorting Intention as a Specific Pro-environmental Behavioral Intention

Waste sorting can be understood as the process of separating waste into a variety of categories within different settings (e.g., the household, tourism destination, and workplace) for efficient waste management, including recycling and reprocessing [7]. This research focuses on tourists’ intentions of separating waste in the heritage destinations. Considering that various types of waste are suitable for recycling and reprocessing, sustainable waste management is a critical strategy for the conservation, reduction and reuse of limited resources on the Earth [41]. For destinations, how tourists dispose of waste during their trip can have a direct effect on the destination environment and public health (both visitors and residents) in the long term. Therefore, waste sorting intention can be understood as a specific type of pro-environmental behavioral intention. Tourists’ participation in waste sorting should be encouraged to achieve the sustainability of environment and tourism. Prior studies documented several types of waste disposal behaviors and intentions, such as waste recycling behavior and binning behavior [21,42]. In the domain of waste sorting, however, existing research mainly involves waste separation in urban or rural households [43,44]. Only a small amount of research has investigated how tourists form intentions to sort waste [7], and much remains unclear about the antecedents of people’s waste sorting intentions in the context of tourism. 

### 2.2. Theory of Planned Behavior (TPB)

As a theoretical derivation of the theory of reasoned action, TPB has been widely employed to explain determinants of a variety of social behaviors, including pro-environmental behaviors [20]. The central tenet of TPB is that an individual’s intention to adopt a particular behavior is the most immediate predictor of the actual behavioral enactment [19]. The key components involving in the intention formation processes are attitudes toward the behavior, subjective norms, and perceived behavioral control [19]. According to TPB, an individual’s strong behavioral intention is driven by one’s favorable attitudes regarding the behavior, normative support for performing the behavior from important others, and volitional control over the particular behavior [45]. 

The utility of TPB has been extensively examined in the research on pro-environmental behaviors and intentions in general and specific terms [46,47,48]. Furthermore, a large amount of existing literature extends the TPB framework with other constructs or theories due to the rationality orientation of TPB [19]. For example, TPB has been extended with the norm activation model to investigate tourists’ intentions to use eco-friendly products instead of single-use plastics [46]. The results indicate that TPB constructs are all relevant in understanding beach visitors’ intentions to use green products. Particularly, TPB has also shown its efficacy in explaining waster sorting intentions [9]. For example, TPB has been adopted to identity factors influencing residents’ engagement in household waste separation in Ecuador [9]. Findings suggest that residents’ environmental attitudes, perceived convenience and subjective norms promote household waste sorting engagement. Furthermore, a few researchers have also extended the TPB framework with social capital to explain the waste sorting intentions from a tourist perspective, with the results supporting the explanatory capacity of TPB [7]. The aforementioned evidence provides valid grounds for this research to explain tourists’ waste sorting intentions with an extended TPB framework. However, the extension of TPB with an individual’s identity perspective and personal normative factors in investigating tourists’ waste sorting intentions has received very limited attention. Based on the TPB framework, the current research endeavors to expand the knowledge on tourists’ waste sorting intentions by adding tourists’ self-identity and moral norms. 

#### Relationships between TPB Constructs and Tourists’ Waste Sorting Intentions 

Attitudes toward the behavior refer to the overall assessment (favorable or negative) of partaking in the behavior [19]. According to TPB, attitudes are shaped based on one’s perceptions of costs and gains of performing the behavior [49]. In the context of tourism, when visitors hold more positive attitudes toward acting in a pro-social manner, they are more likely to adopt sustainable environmental behaviors [45]. As such, if visitors believe that the benefits of separating waste (e.g., sustained environmental quality and satisfactory tourism experience) outweigh the costs (e.g., extra efforts and inconvenience), they will develop more positive attitudes toward waste sorting, which in turn enhance their behavioral intentions. The link between visitors’ attitudes toward the behavior and pro-environmental behaviors and intentions (including waste sorting intentions) has been verified in previous tourism research [7,47,50]. Thus, this research proposed that:

**H1.**  *Attitudes toward the behavior directly and positively affect tourists’ waste sorting intentions.*

According to TPB, an individual’s behavior is also influenced by subjective norms [20]. Subjective norms represent people’s perceptions about the expectations of important people in their lives regarding the behavior and an implicit motive to comply with the perceived social pressure [19]. Tourists are more likely to form stronger intentions to protect the destination environment during their travels when they receive support from relevant individuals such as family, peers, or close friends for adopting environmentally conservative behaviors [20]. Since waste sorting is viewed as a specific pro-environmental behavior, tourists’ waste sorting intentions can also be explained by similar theoretical rationale. Prior empirical studies on environmental behaviors and intentions have verified the effects of tourists’ subjective norms on their intentions to act pro-environmentally [32,51,52]. Evidence in the research of waste disposal behaviors also supports the association between visitors’ subjective norms and waste disposal behaviors and intentions, such as picking up litter in protected areas [51], binning behavior in national parks [53], as well as waste sorting intentions in rural destinations [7]. Consequently, it was hypothesized that:

**H2.**  *Subjective norms directly and positively affect tourists’ waste sorting intentions.*

Perceived behavioral control measures people’s volitional control over their activities related to the impact from external conditions which may promote or deter the performance of these activities [19,54]. That is, when tourists consider that they are equipped with sufficient resources or skills to protect the environment (e.g., disposing of waste properly), they will have a propensity to take responsible environmental actions, such as picking up litter or separating waste. This positive association between perceived behavioral control and tourists’ pro-environmental behavioral intentions has been clearly established in extant tourism research [20,55]. Furthermore, Cao et al. report that perceived behavioral control is positively associated with tourists’ intentions to sort waste in rural destinations [7]. Therefore, the following hypothesis was formulated:

**H3.**  *Perceived behavioral control directly and positively affects tourists’ waste sorting intentions.*

### 2.3. Self-Identity as an Additional Predictor of Tourists’ Waste Sorting Intentions

Although the three attitudinal factors in TPB (i.e., attitudes toward the behavior, subjective norms, and perceived behavioral control) can explain the formation of individual intentions to perform certain behaviors [31], the exclusion of an individual’s socially-relevant factors restricts the predictive power of this rational-based theory [56]. This makes the inclusion of non-rational factors necessary for fully capturing the determinants of an individual’s behaviors [19]. A noteworthy progress in extending TPB involves the addition of self-identity [57]. Self-identity, the central concept of the identity theory, is described as the label attached to oneself [30]. With its roots tracing back to Mead’s symbolic interaction framework [58], the identity theory postulates that self encompasses multiple roles corresponding to one’s positions in the social structure [30,59]. Based on this, the social positions a person holds can influence how they perceive themselves, which in turn motivates them to modify their behaviors in order to maintain consistency with their identity, values, and beliefs. Though no evidence to date has indicated that tourists’ self-identity would influence their waste sorting intentions, considerable empirical research has validated the nexus between one’s self-identity and environmental behaviors and intentions [60,61], including pro-social behaviors amongst tourists [26,62]. Thus, this research proposed the following hypothesis:

**H4.**  *Self-identity directly and positively affects tourists’ waste sorting intentions.*

#### Relationship between Self-identity and Moral Norms

Stemming from the norm activation model [63], moral norms are defined as one’s beliefs of the rightness or wrongness of certain behaviors [64]. They are also referred as personal norms in the literature [42]. As primarily internalized values, moral norms entail beliefs that manifest one’s personal moral commitment to behave in a particular manner [63]. There is strong evidence in the existing literature that demonstrates the important role of moral norms in people’s decision-making processes [65], including the decision to act pro-environmentally [66]. It is suggested that self-identity impacts how individuals perceive their moral responsibility to engage in certain behaviors, particularly when the contradiction between personal interests and those of others is involved in the decision-making process [26]. Prior research also indicates that stronger normative identities are associated with a higher probability of moral sentiments [67], which implies that people’s self-identity may reinforce their moral norms towards performing certain behaviors [63,68]. In other words, when people view themselves as environmental-friendly individuals, they will feel more obligated to take relevant actions for environmental protection, such as sorting waste. For example, self-identity has been found to have a direct and positive effect on personal norms of science teachers in Turkey [39]. Likewise, it is suggested that people with a stronger sense of environmental self-identity tend to hold stronger moral norms in some experimental studies [34]. Accordingly, this research assumed that:

**H5.**  *Self-identity directly and positively affects moral norms.*

### 2.4. Relationship between Moral Norms and Tourists’ Waste Sorting Intentions 

This notion of moral norms represents an individual’s own expectations for a particular type of conduct in a specific circumstance. When activated, they are perceived as moral obligation [69]. Hence, in the context of tourism, tourists with a strong sense of personal norms to behave pro-environmentally will feel duty-bound to act accordingly. The harm of reckless waste disposal to the environment can evoke their moral obligations to sort waste in the destinations. Prior empirical studies have indicated that moral norms affect people’s pro-environmental behaviors and intentions, such as tourists’ binning behavior [42], tourists’ waste reduction intentions and behaviors [70,71], students’ recycling behavior [38], and residents’ waste sorting intentions [72]. For instance, moral norms are confirmed as the most significant antecedent of tourists’ pro-environmental binning behaviors in ecotourism [42]. Similarly, in the investigation of waste separation behavior of Malaysian residents, Razali et al. note that the major factor influencing residents’ internal motive to separate household waste is their moral norms [73]. Findings of these studies offer solid support for this reasoning. Thus, this research proposed the following hypothesis:

**H6.**  *Moral norms directly and positively affect tourists’ waste sorting intentions.*

#### The Mediating Role of Moral Norms between Self-identity and Tourists’ Waste Sorting Intentions

Even though previous empirical evidence shows that self-identity directly affect pro-environmental behaviors and intentions, it remains inconclusive why people are driven to act in a certain manner that corresponds to their self-identity [62]. The existing literature suggests that self-identity can predict one’s sustainable behaviors without external incentives, which may indicate the involvement of an obligation-based intrinsic motivation [34]. As one’s perception of moral obligation to adopt or refrain from particular actions is referred as moral norms [63], moral norms thus fall under the umbrella of obligation-based motives. To this end, moral norms may act as the mediator that connects self-identity and pro-environmental behaviors. For example, an individual’s self-identity is documented to positively link with their moral norms, which subsequently predicted people’s intentions to engage in the Earth Hour Campaign in Hong Kong [26]. Similarly, Lee et al. (2021) confirm the sequential causal relationship between tourists’ environmental self-identity, moral norms, and pro-environmental behaviors in a context of ecotourism [69]. Therefore, it was formulated that:

**H7.**  *Moral norms mediate the positive effect of self-identity on tourists’ waste sorting intentions.*

**Figure 1:**
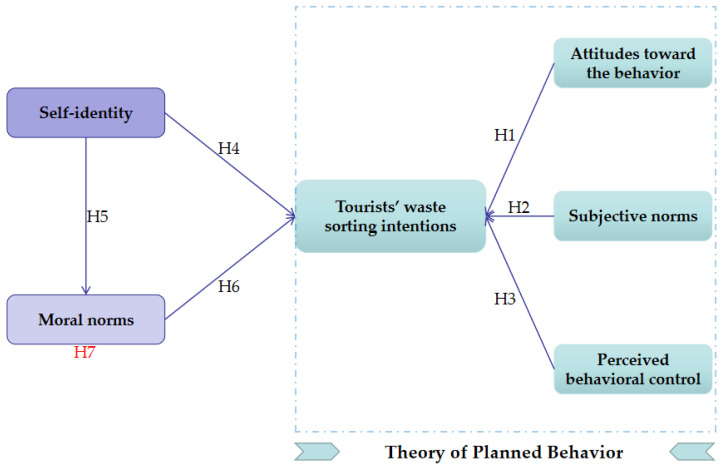
The proposed conceptual model of this research. Note: H1−H6 are direct hypotheses, H7 is an indirect hypothesis.

Drawing on the aforementioned discussions and review of the previous literature, this research proposed an extended TPB framework enriched with self-identity and moral norms for predicting tourists’ waste sorting intentions. Figure 1 presents the proposed conceptual framework of the present study. 

## 3. Methods 

The structural equation modeling (SEM) approach was adopted in this research to conduct the data analysis. After proposing the conceptual model presented in Figure 1, we conducted a pretest of the measurements of all constructs involved to ensure the reliability and validity of the scales. With the date achieved from the field survey, the confirmatory factor analysis was carried out in AMOS to determine the overall fit between the measurement model and data. Then, the structural model was tested to ensure a good fit. The final steps include the analysis of direct and indirect paths, as well as the explanatory power test. 

### 3.1. Measurement

Each item in the measurement was measured with five-point Likert scales, ranging from 1 (strongly disagree) to 5 (strongly agree). The scale for measuring attitudes towards the behavior (ATT) was adopted from Zheng et al. (e.g., ‘During this trip, I thought waste sorting was a wise behavior.’), so was the scale for perceived behavioral control (PBC) (e.g., ‘During this trip, whether or not I sorted waste was up to me.’) [48]. Subjective norms (SN) was adapted from the scales developed by Li et al. (e.g., ‘During this trip, those important to me thought I should sort waste.’) [56]. Three items evaluating self-identity (SI) were adapted from the scale developed by Barbarossa et al. (e.g., ‘During this trip, I thought of myself as someone who was concerned about waste sorting.’) [74]. The three-item scale developed by Qiu et al. was applied to measure moral norms (MN) (e.g., ‘I felt a moral obligation to sort waste when traveling in this destination.’) [15]. Three items were adapted from Meng and Choi to measure tourists’ waste sorting intentions (TWSI) (e.g., ‘I intend to sort waste at this destination.’) [75]. The item scales were adjusted properly according to the specific research context. The detailed measurements of all constructs were listed in the Appendix A.

### 3.2. Pretest of Measurements

The initial version of the questionnaire was drafted in English and translated into Chinese. Then a back-translation approach was adopted to ensure accuracy [76]. The content validity was evaluated by inviting three tourism researchers and two managers in tourism destinations. Then, a pilot study was conducted by recruiting 100 tourists who visited the research site to ensure the reliability and validity of the scales. As the preliminary results indicated, the Cronbach’s alpha was above the recommended cutoff value of 0.70, whereas the standard factor loadings were higher than 0.50, suggesting acceptable reliabilities and validity [77,78].

### 3.3. Data Collection 

**Figure 2:**
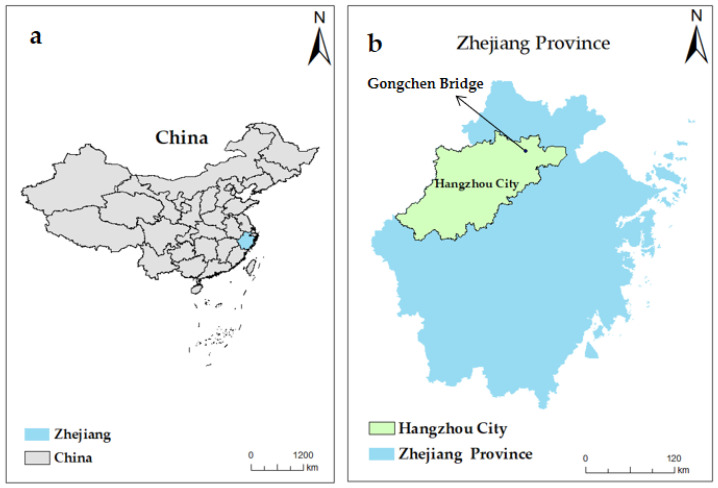
(**a**) The geographical location of Zhejiang Province in the People’s Republic of China; (**b**) the geographical location of the study site in Hangzhou, Zhejiang Province. Note: Gongchen Bridge, where the field survey was conducted, is an iconic scenic spot of the Grand Canal Scenic Area in Hangzhou.

The data were collected from a heritage destination in China named the Beijing–Hangzhou Grand Canal (Hangzhou section). With its earliest history dating back to the 5th century BC, the Grand Canal runs through eight provinces from the capital Beijing to Hangzhou. As the longest and oldest canal in the world, it constitutes a vast inland waterway system that that facilitated the supply of rice, centralized management of the region, and transportation of troops [79]. It has made significant contributions to the stability and flourishing of the nation’s economy. This canal remains in use today as a vital means for communication between the north and south of the country. In June 2014, the Grand Canal was listed as a World Heritage Site by the World Heritage Committee [79]. Though the official visitation number of the Grand Canal is not available online, it was reported that the visitation of the Grand Canal Scenic Area reached over 400,000 person-time during the 7-day National Day Holiday in 2018 [80]. Today, it is still a popular and iconic heritage destination for people visiting the Grand Canal and the city. Thus, this heritage destination was selected as the study site for the present research. Figure 2 presents the geographical location of the study site.

The epidemic control and prevention measures had not been lifted when the survey was conducted from October to November 2022. Therefore, the majority of the respondents were Chinese domestic tourists. Four groups consisting of one researcher and one assistant conducted the field survey with the convenience sampling approach. The respondents were provided with a brief introduction and instructions before filling in the questionnaires. If people showed reluctance or were unqualified, the researchers would approach the next available participant. A total of 450 questionnaires were collected with 403 valid responses, showing an 89.6% effective response rate. As Nunnally (1967) recommended, 403 valid responses are sufficient since the number is higher than the minimum size of 190 (ten times of the total number of all items) [81]. The participants demonstrated a balanced gender ratio (48.1% of male and 51.9% of female); the respondents’ demographic profile was illustrated in Table 1. Both the values of univariate skewness statistics (−0.632 to −0.198) and kurtosis statistics (−0.679 to 1.295) fell within the acceptable range [82,83]. 

## 4. Results 

### 4.1. Common Method Bias Analysis

A common method bias (CMB) test is required in survey-based study, particularly when the data is collected from the same source [84,85]. First, Harman’s single-factor test was performed in SPSS. The results showed that no single factor explained the covariance of more than 50% (the first factor explaining 48.203% of the total variance) [86]. Then, confirmatory factor analysis was conducted to access if all of the variances could be explained by a common latent factor. The proposed model outperformed the common factor model (Δχ2(15) = 1733.855, *p* < 0.001). Hence, it is claimed that CMB was not a pervasive problem for this study [86].

### 4.2. Measurement Model Analysis

We performed confirmatory factor analysis (CFA) to evaluate the measurement model in this study with AMOS [87,88]. Findings from this study suggest that the measurement model fits the data well, with χ^2^/df, RMR, RMSEA being 1.200, 0.012, and 0.022, respectively. GFI, NFI, IFI, TLI, CFI, and SRMR are 0.960, 0.970, 0.995, 0.993, 0.995, and 0.027, respectively.

According to the results in Table 2, the Cronbach’s alphas ranged from 0.824 to 0.915, and the composite reliability spanned from 0.828 to 0.916, which indicated that the measurement model had acceptable internal reliability [89]. Regarding convergent validity, the standardized factor loadings ranged from 14.287 to 24.288; the average variance extracted (AVE) from 0.617 to 0.783; and the composite reliability from 0.828 to 0.916, indicating a satisfied convergent validity [90,91]. 

The discriminant validity reflects the degree to which a specific construct is distinct from other constructs [92]. We access the discriminant validity by comparing the square root of each construct’s AVE with the correlations between corresponding latent constructs validity [90]. The results in Table 3 indicated that the square root of AVE values ranged from 0.785 to 0.885, exceeding the construct correlation values (ranging from 0.523 to 0.639). As a result, there was adequate discriminant validity. Therefore, we established both the reliability and validity of the measurement model. 

### 4.3. Structural Model Analysis

Confirmatory factor analysis was used to assess the measurement model fit, as well as the constructs’ validity and reliability before examining the direct hypotheses with structural equation modeling (SEM) [93]. According to the fit dices (χ2/df = 1.603, RMR = 0.028, RMSEA = 0.039, GFI = 0.945, NFI = 0.959, IFI = 0.984, TLI = 0.980, CFI = 0.984, SRMR = 0.062), there is a good fit for the proposed model. Table 4 presents the detailed direct hypothesis test results. 

**Figure 3:**
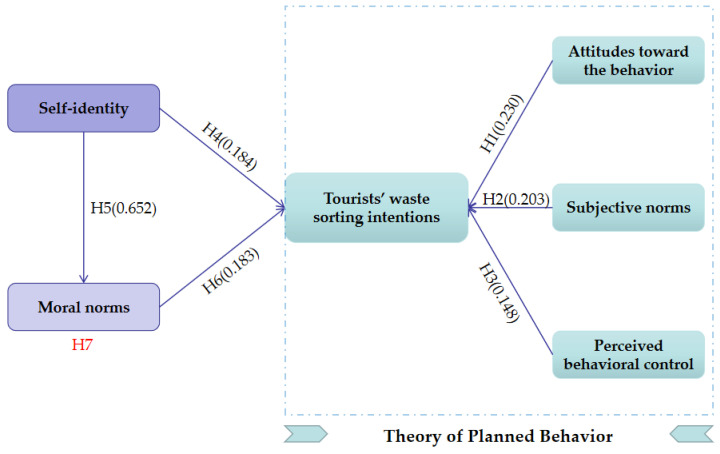
AMOS output results of the model in this research.

According to the results, there were direct and positive relationships between the TPB variables (i.e., ATT, SN, PBC) and TWSI (β_ATT_ = 0.230, *p* < 0.001; β_SN_ = 0.203, *p* < 0.001; β_PBC_ = 0.148, *p* < 0.05), suggesting H1, H2, and H3 were supported. SI was directly and positively linked to MN (β =0.652, *p* < 0.001) and TWSI (β = 0.184, *p* < 0.05), respectively, which confirmed H4 and H5. Lastly, there was a direct and positive relationship between MN and TWSI (β = 0.183, *p* < 0.001), indicating H6 was supported. Therefore, all six direct hypotheses proposed in this research were supported. Figure 3 exhibits the AMOS output results.

### 4.4. Mediating Effect Analysis

There were several methodologies for testing the mediation effect, including the causal steps method, the Sobel test, and the bootstrapping approach. Developed by Baron and Kenny, the causal steps method was widely applied [94]. However, there are two issues with this technique. First, simulation research has found that this is one of the least effective techniques for testing mediation effect [95,96]. Second, this technique does not account for the degree of the mediation effect [97], which prevents it from being compatible with frameworks that have inconsistent mediation [98]. Often used in addition to the causal steps technique [99], the Sobel test is built on the premise of normal sampling distribution of the indirect effect. However, the sample distribution of *ab* usually shows asymmetry, nonzero skewness and kurtosis [100]. Since bootstrapping can prevent a significant Type I error rate due to the breach of the normal distribution, it is deemed to be preferable to the traditional Sobel test [101]. A considerable number of recent studies have used the bootstrapping approach to assess the mediating effect [82,102,103,104]. 

Accordingly, this research applied the AMOS’s bootstrapping analysis to examine the mediating effect with 5000 iterations and a 95% bias-corrected confidence interval [105]. Table 5 suggests that there is a significant mediating effect of SI on TWSI via MN (β = 0.119; CI = (0.044, 0.205); *p* < 0.01). 

### 4.5. Explanatory Power of the Conceptual Model 

The explanatory power of a model can be evaluated by the *R*^2^ values of its endogenic variables. The threshold value to indicate the large, medium, and small effect of the model were 0.25, 0.09, and 0.01, respectively [106]. According to the squared multiple correlations (SMC = *R*^2^) in Table 6, theory of planned behavior (i.e., M0), the self-identity model (i.e., M1), and the integrated model (i.e., M2) explained 52.9%, 45.7%, and 56.3% of the variance for TWSI, which demonstrated the superior explanatory power of the integrated model comparatively. 

## 5. Conclusions, Discussion, and Implications 

### 5.1. Conclusions and Discussion 

Tourists separating waste in heritage sites can essentially help sustain the environment, tourism development, as well as territorial development in the long term [6,107]. By taking self-expressive and personal normative factors into consideration, this study extended the TPB model to investigate the roles of self-identity and moral norms in explaining tourists’ intentions to separate waste in the heritage destinations. Results of the SEM analysis indicated that all the proposed hypotheses in this study were confirmed.

The results indicated that three TPB constructs (i.e., attitudes toward the behavior, subjective norms and perceived behavioral control) all positively and directly affected tourists’ waste sorting intentions, confirming H1, H2, and H3. These results are aligned with prior studies by Gao et al. and Cao et al. in investigating tourists’ pro-environmental behavioral intentions and specific behavioral intentions, respectively [7,55]. This study thus further validated the explanatory value of TPB in explaining tourists’ pro-environmental behavioral intentions and waste sorting intentions. Specifically, it indicates that tourists will form intentions to classify waste if they hold more positive attitudes toward waste sorting. A higher level of social pressure from others or perception of sufficient knowledge or skills for adopting the behavior will lead to the same results. Accordingly, TPB is reconfirmed to be a feasible theoretical foundation for predicting tourists’ waste sorting intentions in the heritage tourism setting [31]. 

Furthermore, we have also investigated how tourists’ self-identity influenced their waste sorting intentions and moral norms. The results suggested that tourists’ self-identity was positively associated with their waste sorting intentions (H4 was supported). This is consistent with previous endeavors of Chan et al. and Lee et al. whose studies indicate that one’s self-identity is crucial in promoting their pro-environmental behaviors or intentions related to one’s certain specific label in the social structure [26,62]. Unlike the emphasis on the effects of tourists’ social network on their behavioral intentions [7], this results underscored the importance of self-expressive factor (i.e., self-identity) in triggering their specific pro-environmental behavioral intentions. In addition, our results also suggested that H5 was confirmed, which further verified a positive effect of self-identity on moral norms. This is in line with findings of prior studies [34,39], which found that visitors’ moral norms to behave sustainably can be provoked when they define themselves as pro-environmental people.

Lastly, we examined the association between tourists’ moral norms and their waste sorting intentions. The results showed that moral norms were positively related to visitors’ waste sorting intentions (i.e., H6 was confirmed). This agrees with the findings of the existing literature on how tourists’ moral considerations influence their pro-environmental behavioral intentions [42], as well as a series of sustainable waste disposal intentions in multiple settings [38,71,73]. Furthermore, the mediating effect analysis was conducted to test the indirect path from self-identity to tourists’ waste sorting intentions via their moral norms. The results confirmed the mediating effect, i.e., H7 was supported. This is consistent with the findings of recent studies on pro-environmental behavioral intentions [40,69]. This means that one’s belief of ‘who I am’ drives them to change their moral sentiments and subsequently their behavioral intentions. Specifically, the extent to which tourists see themselves as waste sorters enhances their moral obligations, which further develops their intentions to sort waste for maintaining their own self-identity.

### 5.2. Theoretical Contributions 

Based on TPB, this current research probed the factors that help the formation of tourists’ waste sorting intentions by adding self-identity and moral norms. In this regard, the findings of this study contributed to the research on pro-environmental behavior and behavioral intention in the following ways. 

This research made an effort to expand our knowledge of how TPB can be integrated with the identity theory and moral norms to explain one’s intentions of waste sorting in a context of tourism. This study constituted a robust conceptual theoretical framework with several variables drawn from multiple theories or models, e.g., the identity theory and the norm activation model. To be specific, the key elements of this conceptual model include volitional and non-volitional factors in TPB, the self-expressive factor (i.e., self-identity) in the identity theory, and personal normative factors (i.e., moral norms) in the norm activation model. There have been a few attempts to extend TPB with other theories or constructs in the research on tourists’ waste disposal behaviors and intentions, including tourists’ binning behaviors [42], waste reduction intentions [71], and waste sorting intentions [7]. However, the primary focal point of these studies is on the impact of personal normative factors or social network. However, there is oversight of tourists’ identity perspective in the extended TPB framework. Therefore, the present study extended the existing literature with the addition of the self-identity and deepened our understanding of the formation of waste sorting intentions amongst tourists. 

Furthermore, the findings contributed to the current body of knowledge through investigating the role of moral norms in mediating the effect of self-identity on tourists’ waste sorting intentions. The importance of self-identity and moral norms in stimulating tourists’ pro-environmental behavioral intentions has been separately acknowledged by previous research [42,60,62]. However, the interrelationship between self-identity and moral norms still needs more empirical evidence in explaining specific pro-environmental behavioral intentions [26], particularly waste sorting intentions in the context of tourism. Our results confirmed the mediating role of personal moral norms, which provided more insights into the transformation from one’s self-identity into waste sorting intentions. It corroborates the views in previous studies that one’s self-identity reinforces their moral obligations, which in turn impose impact on corresponding behaviors [36,37]. To sum up, this finding furthered our understanding of the sequential causal relationship between self-identity, moral norms, and waste sorting intentions in the context of tourism. 

### 5.3. Managerial Implications 

TPB was extended with self-identity and moral norms to understand the development of tourists’ waste sorting intentions in this research. Apart from the aforementioned theoretical implications, the findings also provided a number of practical implications which may help destination managers and other public agencies encourage tourists to engage in waste sorting. 

First, similar to other research findings [8,9], three TPB constructs (i.e., attitudes toward the behavior, subjective norms, and perceived behavioral control) were confirmed to be effective in predicting tourists’ waste sorting intentions. This implies that visitors should be informed that their actions do make a difference in maintaining the environmental integrity of the destinations. For example, social media influencers or popular public figures can be invited to participate in producing the promotion video of the destination. Their advocacy of environmental protection or waste sorting may raise tourists’ awareness of proper waste disposal. Volunteers can be recruited to assist waste sorting at the destination. Prosocial actions of the volunteers may inspire tourists and improve their perception of social pressure. To improve tourists’ sense of volitional control over waste sorting, destination managers and local administrative agencies can collaborate to ease the difficulties of waste sorting. Feasible measures can include displaying posters advocating waste sorting and distributing more garbage bins in conspicuous places.

Second, the direct and indirect routes from tourists’ self-identity to their waste sorting intentions underlined the significant predictive ability of self-identity in shaping tourists’ behavioral intentions. This suggested that useful interventions could be developed to facilitate the establishment or enhancement of tourists’ self-identity as waste sorting travelers. Since tourists’ self-identity reflects how they position their own social roles, the destination and public agencies could cooperate to improve tourists’ level of self-identity via various forms. For instance, the public should be guided to sort waste in the unhabitual environment through environmental education campaigns by public agencies. For destinations, publicity videos, waste sorting posters, or reminders should be displayed at different venues to imperceptibly influence tourists’ self-image. Overall, the goal of these strategies is to cultivate tourists’ perception of being the type of people who intend to dispose their garbage properly. 

Lastly, considering the critical role of people’s internalized values (i.e., moral norms) in forming the intentions of waste sorting [73], effective communication strategies should be developed to reinforce their moral obligations to sort waste. The role of moral norms as direct determinants of visitors’ waste sorting intentions and the mediator between self-identity and behavioral intentions provide solid ground for this managerial implication. As people’s view of the world are the foundation of their moral obligations [63], the purpose of these communication tactics is to influence or change people’s view of and their relationship with the world. For example, brochures or information in other forms (e.g., audio or visual) should be available which enable visitors to realize the damages of irresponsible waste disposal inflicted on the environment. When tourists become aware of the impact of their behaviors on the environment, they may feel morally obligated to preserve the environment for harmonious coexistence. 

## 6. Limitations and Future Research Directions 

Despite the potential contributions and implications to the academic community and destination managers, there were some limitations in this research. First, this research employed the SEM method for empirical analysis. However, an increasing number of scholars begin to apply more diverse approaches in tourism research. For example, a growing body of literature has turned to conducting experiments [108,109]. Others have tried mixed methods that combine with fuzzy set qualitative comparative analysis, focus groups, or in-depth interviews [110,111,112]. Such methodologies can be the alternatives for future research. Second, the field survey was conducted at only one site due to the epidemic prevention policies implemented (between October and November of 2022). In order to improve the stability and applicability of the conceptual model, future research should consider two or more case studies for cross-validation [113]. Lastly, since tourists may behave differently at home and destinations [10], more empirical evidence is required to validate the influence of the habitual and unhabitual environments. Therefore, future work could consider the comparison of people’s waste sorting intentions at homes and destinations. 

## Figures and Tables

**Table 1:** Profile of the respondents.

Variable	Category	n	(%)
Gender	Male	194	48.1
	Female	209	51.9
Age	<25	96	23.8
	25–34	115	28.5
	35–44	91	22.6
	45–59	66	16.4
	≥60	35	8.7
Education	Less than high school/technical school	32	7.9
	High school/technical school	52	12.9
	Diploma education	118	29.3
	Undergraduate degree	152	37.7
	Graduate degree and above	49	12.2

Note: n = 403.

**Table 2:** Results of the measurement model.

Construct	Loading	T-Values	CR	AVE	Cronbach’s Alphas
ATT			0.880	0.647	0.879
ATT1	0.762	17.316			
ATT2	0.788	18.140			
ATT3	0.816	19.058			
ATT4	0.848	—			
SN			0.888	0.726	0.887
SN1	0.852	21.184			
SN2	0.826	20.284			
SN3	0.877	—			
PBC			0.828	0.617	0.824
PBC1	0.741	14.287			
PBC2	0.843	15.790			
PBC3	0.768	—			
SI			0.882	0.714	0.877
SI1	0.857	18.359			
SI2	0.890	18.956			
SI3	0.784	—			
MN			0.909	0.769	0.909
MN1	0.863	22.111			
MN2	0.908	23.770			
MN3	0.859	—			
TWSI			0.916	0.783	0.915
TWSI1	0.889	23.808			
TWSI2	0.900	24.288			
TWSI3	0.866	—			

Note: ATT = attitudes toward the behavior; SN = subjective norms; PBC = perceived behavioral control; SI = self-identity; MN = moral norms; TWSI = tourists’ waste sorting intentions; CR = composite reliability; AVE = average variance extracted.

**Table 3:** Results of discriminant validity.

Construct	ATT	SN	PBC	SI	MN	TWSI
ATT	[0.804]					
SN	0.639	[0.852]				
PBC	0.573	0.544	[0.785]			
SI	0.523	0.551	0.580	[0.845]		
MN	0.581	0.533	0.579	0.613	[0.877]	
TWSI	0.639	0.621	0.595	0.605	0.610	[0.885]

Note: ATT = attitudes toward the behavior; SN = subjective norms; PBC = perceived behavioral control; SI = self-identity; MN = moral norms; TWSI = tourists’ waste sorting intentions.

**Table 4:** Structural model assessment and hypothesis test results.

Hypotheses	Path	Standardized Coefficient	T-Value	Results
H1	ATT→TWSI	0.230	3.758 ***	Supported
H2	SN→TWSI	0.203	3.369 ***	Supported
H3	PBC→TWSI	0.148	2.425 *	Supported
H4	SI→TWSI	0.184	2.512 *	Supported
H5	SI→MN	0.652	11.941 ***	Supported
H6	MN→TWSI	0.183	3.297 ***	Supported

Note: * *p* < 0.05, *** *p* < 0.001. ATT = attitudes toward the behavior; SN = subjective norms; PBC = perceived behavioral control; SI = self-identity; MN = moral norms; TWSI = tourists’ waste sorting intentions.

**Table 5:** Mediation test results.

Mediating Hypothesized Path	Indirect Effects	Lower	Upper	*p*-Value	Results
H7: SI→MN→TWSI	0.119	0.044	0.205	0.004	Supported

Note: SI = self-identity; MN = moral norms; TWSI = tourists’ waste sorting intentions.

**Table 6:** Model comparison test results.

Model Category	*R*^2^: TWSI
M0: TPB	0.529
M1: the self-identity model (including SI and MN)	0.457
M2: M0 + M1	0.563

Note: TPB = theory of planned behavior; SI = self-identity; MN = moral norms; TWSI = tourists’ waste sorting intentions.

## Data Availability

Not applicable.

## Appendix A

**Table A1:** Detailed measurements of all variables and literature sources.

Construct	Item	Source
Attitude toward the behavior (ATT)	ATT1 During this trip, I thought waste sorting was a wise behavior.	[48]
	ATT2 During this trip, I thought waste sorting was a valuable behavior.	
	ATT3 During this trip, I thought waste sorting was a necessary behavior.	
	ATT4 During this trip, I thought waste sorting was a beneficial behavior.	
Subjective norms (SN)	SN1 During this trip, those important to me thought I should sort waste.	[56]
	SN2 During this trip, those important to me expected me to sort waste.	
	SN3 During this trip, those important to me were delighted if I sorted waste.	
Perceived behavioral control (PBC)	PBC1 During this trip, whether or not I sorted waste was up to me.	[48]
	PBC2 During this trip, I was capable of sorting waste.	
	PBC3 During this trip, I was confident that if I wanted, I could sort waste.	
Self-identity (SI)	SI1 During this trip, I thought of myself as someone who was concerned about waste sorting.	[74]
	SI2 During this trip, I thought of myself as a “waste sorting” consumer.	
	SI3 During this trip, I would describe myself as a waste sorting conscious consumer.	
Moral norms (MN)	MN1 I felt a moral obligation to sort waste when traveling in this destination.	[15]
	MN2 Because of my own values/principles, I felt an obligation to sort waste when traveling in this destination.	
	MN3 I felt that I should sort waste when traveling in this destination.	
Tourists’ waste sorting intentions (TWSI)	TWSI1 I intend to sort waste at this destination.	[75]
	TWSI2 I am willing to sort waste at this destination.	
	TWSI3 I am planning to sort waste at this destination.	
